# Bisphosphonate Drug Holidays: Evidence From Clinical Trials and Real‐World Studies

**DOI:** 10.1002/jbm4.10629

**Published:** 2022-05-24

**Authors:** Mawson Wang, Yu‐Fang Wu, Christian M. Girgis

**Affiliations:** ^1^ Department of Diabetes and Endocrinology Westmead Hospital Westmead NSW Australia; ^2^ Faculty of Medicine and Health University of Sydney Sydney NSW Australia; ^3^ Department of Endocrinology Royal North Shore Hospital St Leonards NSW Australia

**Keywords:** ATYPICAL FEMUR, FRACTURE, BISPHOSPHONATES, BONE MINERAL DENSITY, BONE TURNOVER MARKERS, DRUG HOLIDAY, FRACTURE, OSTEOPOROSIS

## Abstract

Bisphosphonates (BPs) are commonly used in the treatment of osteoporosis and are effective in the prevention of fragility fracture. Long‐term use has been associated with the development of atypical femur fractures (AFFs) and osteonecrosis of the jaw (ONJ). Drug holidays seek to reduce the risk of insufficiency fractures (AFFs) while maintaining durable effects of long‐term treatment in the prevention of fragility fracture. Guidelines suggest that BP drug holidays be considered after 3 to 5 years. However individual factors impacting this decision and outcomes are unclear. This review examines key factors in the planning of a safe BP drug holiday and surrogate markers of fracture risk in patients discontinuing treatment. Fifteen randomized control trials and 19 real‐world studies were included, including nationwide prospective studies from several countries. Increases in bone turnover markers (BTMs) and reductions in bone mineral density (BMD) were generally observed during BP drug holidays. Resurgent bone turnover was problematic in high‐risk patients in whom fractures recurred as early as 12 months following a drug holiday. Risk factors for holiday‐related fractures included older age, low hip BMD, underweight, low medication adherence, and prevalent/incident fractures. Zoledronic acid conferred the most durable reduction in fractures, particularly after six annual infusions. Five years of alendronate was insufficient in preventing vertebral fractures in high‐risk patients embarking on a drug holiday. Relatively faster offset of antiresorptive effect was seen in risedronate users with more frequent fractures than alendronate during a drug holiday. Studies directly counterbalancing effects of long‐term treatment on AFF risk versus drug holiday outcomes in the same population were lacking. In the absence of persistently high fracture risk and following a specific treatment duration dependent on the BP used, drug holidays are safe and mitigate the risk of AFF. However, anti‐resorptive effects diminish over time; ongoing monitoring and careful planning of BP resumption is necessary. © 2022 The Authors. *JBMR Plus* published by Wiley Periodicals LLC on behalf of American Society for Bone and Mineral Research.

## Introduction

Osteoporosis is a prevalent condition affecting more than 200 million people worldwide.^(^ ^1^ ^)^ An estimated one in three women and one in five men over the age of 50 years will experience an osteoporotic fracture in their lifetime.^(^ ^1^ ^)^ The personal and economic burden of fragility fractures is considerable, with subsequent disability, pain, loss of independence, and increased mortality in affected patients. As the population continues to age and lifespan increases, the incidence of osteoporosis and fragility fractures will increase in the years ahead, becoming a formidable public health issue.

BPs have been in clinical use for more than 30 years and are effective agents in the treatment of osteoporosis. Robust clinical trial data and years of clinical experience support the established effects of BPs in increasing bone mineral density (BMD), reducing fragility fractures, and reducing osteoporosis‐related morbidity.^(^ ^2^, ^3^ ^)^ Additional effects in reducing all‐cause mortality, skeletal‐related events in cancer patients, and possibly cancer diagnoses in particular populations are under examination.^(^ ^4^, ^5^ ^)^

Upon administration, BPs are rapidly cleared from the circulation and bind avidly to exposed surface of hydroxyapatite crystals in bone.^(^ ^6^ ^)^ By becoming incorporated into sites of active bone remodeling, BPs are then available for local release and internalization by active osteoclasts at resorption pits.^(^ ^7^ ^)^ Here, BPs exert multiple actions, including induction of an adenosine triphosphate analogue to induce osteoclastic apoptosis, inhibition of farnesyl diphosphonate synthase (FPPS) leading to inhibition of osteoclast function, and reduced recruitment of osteoclasts.^(^ ^6^, ^8^, ^9^, ^10^ ^)^

A unique feature of the BP drug class is durability of effect beyond their period of use. This relates to the prolonged binding to mineral matrix, endowed by their strong affinity to calcium ions and the potential “recycling” of BPs, which once released at active resorption pits, reattach to adjacent regions of bone mineral.^(^ ^7^, ^11^ ^)^

BPs exhibit differences in affinity for bone mineral and inhibition of FPPS due to distinct structures of the R1 and R2 side‐chains, with zoledronic acid appearing to have the highest skeletal binding affinity, followed by alendronate and risedronate.^(^ ^7^, ^12^ ^)^ The skeletal halflife of BP may be prolonged, in some cases beyond a decade, and relates to baseline degrees of bone turnover, BP‐specific binding affinity, and duration of use.^(^ ^13^, ^14^ ^)^ On the other hand, denosumab, a fully human monoclonal antibody, exerts reversible effects on receptor activator of NF‐κB ligand (RANKL) inhibition with transient inhibition of osteoclast‐mediated bone resorption and risk of vertebral fractures with discontinuation.^(^ ^15^, ^16^, ^17^ ^)^ Reversible effects are also seen with osteoanabolic agents, including the sclerostin inhibitor romosozumab and parathyroid hormone analogues such as teriparatide, which promote the transient recruitment of osteoblasts with new bone matrix formation.^(^ ^18^ ^)^

Due to the inhibition of osteoclastic activity and long residence time in bone, there have been concerns that prolonged treatment with BPs can lead to critical suppression of bone remodeling, microfracture accumulation, and the development of insufficiency fractures.^(^ ^7^, ^19^, ^20^ ^)^ In recent years there is emerging evidence of a duration‐dependent association between BP use and atypical femur fractures (AFFs), with the risk increasing substantially beyond 5 to 8 years of treatment and returning to baseline postcessation.^(^ ^21^, ^22^, ^23^, ^24^, ^25^ ^)^ AFF frequency increases with increased duration of BP treatment, rising from 2.5 AFFs per 10,000 person‐years at ≤5 years of BP use up to 13.1 per 10,000 person‐years at ≥8 years.^(^ ^25^ ^)^

Osteonecrosis of the jaw (ONJ), another adverse event associated with BP use, typically occurs after invasive dental procedures and in higher‐dose BP regimens such as for skeletal metastases and myeloma.^(^ ^10^ ^)^ The incidence of ONJ in the setting of osteoporosis treatment is substantially lower, at around 1 in 10,000 to 100,000 patient‐years compared to 1 to 10 per 100 in patients receiving high‐dose regimens.^(^ ^26^ ^)^ ONJ results from a convergence of factors including local effects of gum/osseous trauma, infection, and vascular mediators, including dysregulation of vascular endothelial growth factor (VEGF), which together, with effects of prolonged BP use, culminate in local bone necrosis.^(^ ^10^ ^)^ Although the association between BPs and the development of AFF increases proportionately with duration of treatment, cumulative high‐dose BP delivered over a short treatment period in a susceptible individual is the typical precursor to ONJ.^(^ ^2^, ^26^ ^)^

The medical management of chronic disease relies on continued adherence to pharmacotherapy. However, a treatment period with BPs affords antiresorptive effects that persist after discontinuation, leading to the concept of a BP drug holiday. The goal of a BP drug holiday is to reduce the risks of complications from prolonged antiresorptive therapy at a time when clinically significant and durable anti‐fracture effects have been achieved.

After how many years of treatment should patients on BP be advised to embark on a drug holiday? What are some of the characteristics of a “safe” BP drug holiday in regard to timing, duration, and resumption of therapy? Are surrogate markers of fracture risk helpful in determining in whom, when, and for how long a BP drug holiday should be undertaken? The present review examines data from long‐term clinical trials and real‐world studies, piecing together answer to these clinically important questions. The answers are complex and contingent on a range of individual factors, including the underlying severity and chronicity of the patient's microstructural bone deficit, their fracture profile, the antiresorptive potency of the particular BP used, and the specific risk of an AFF for that individual.

## Methodology

A literature search was conducted to source randomized controlled trials (RCTs) and real‐world studies evaluating the effect of BP discontinuation on BMD, bone turnover markers (BTMs), and fractures. The databases searched include PubMed, Ovid/Medline, Embase, and Google Scholar. Search terms used included osteoporosis, BP drug holiday, BP discontinuation, BP withdrawal, fracture, bone mineral density, bone turnover markers, randomized control trial, real world study, cohort study, population based study, and observational study.

RCTs included patients who had received BP therapy for at least 2 years, with both long‐term treatment and discontinuation arms, allowing comparison of outcomes in BMD, BTMs, and/or fracture risk. A proportion of real‐world studies compared patients on a BP drug holiday with a cohort of persistent BP users.

**Fig. 1:**
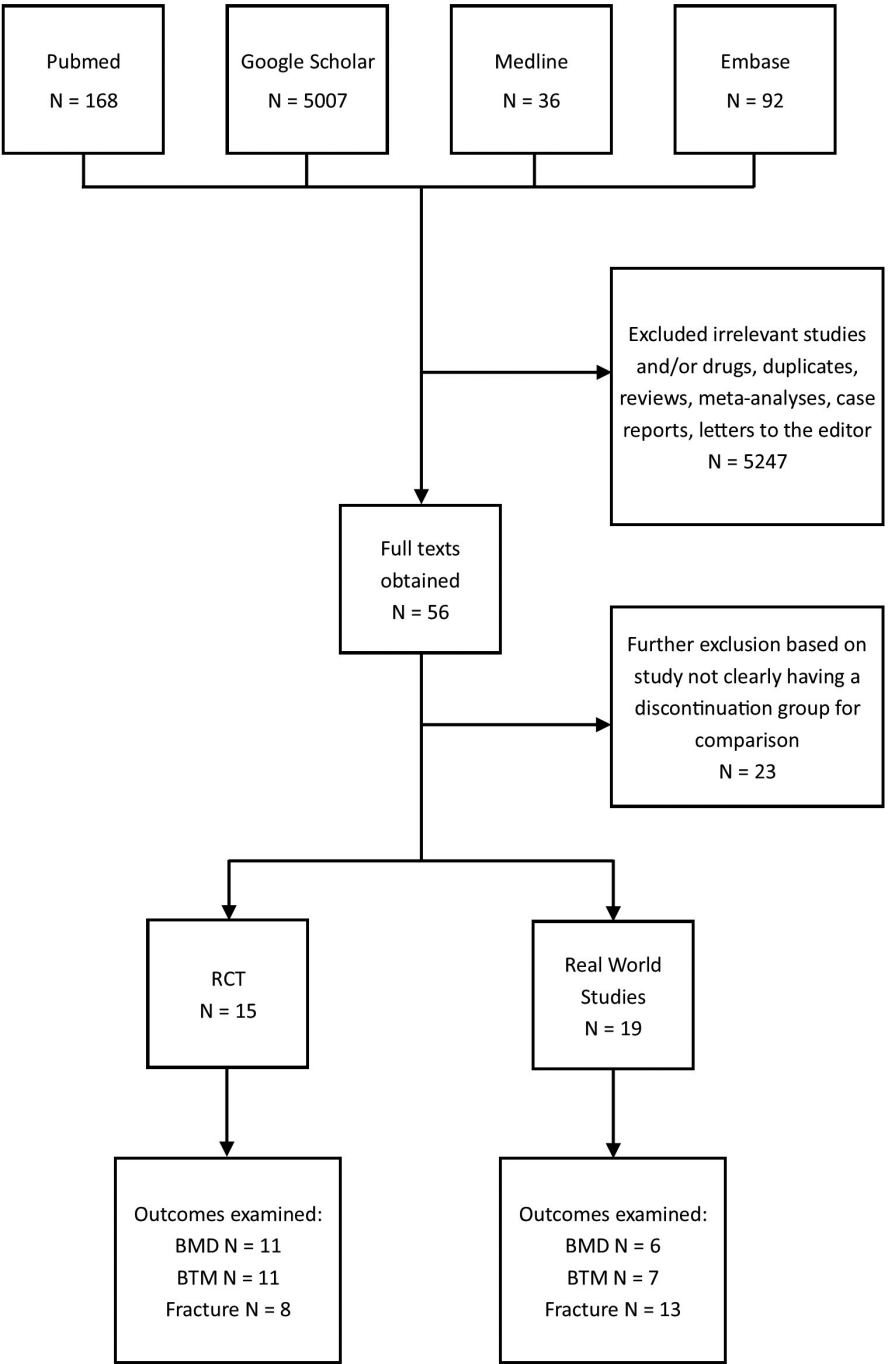
Review of studies.

The literature search yielded 5303 studies, of which the majority were excluded based on lack of relevance, the majority lacking outcome data following discontinuation of BP, or the use of drugs other than BP during the study period. Others were excluded due to the type of study and duplicates (see Fig. 1). We included two denosumab studies with a BP arm that could be analyzed independently from denosumab. Article title and abstracts were screened, followed by full‐text review if deemed suitable for the study.

We identified 15 RCTs as suitable for this review, which examined subjects on currently prescribed nitrogen‐containing BPs (zoledronic acid: four trials, alendronate: nine trials, risedronate: two trials). With regard to outcome measures, 11 studies included data on BMD, 11 on BTMs, and eight included data on fractures. There were 19 suitable real‐world studies including three studies examining alendronate; the remaining studies included patients on alendronate, risedronate, ibandronate, pamidronate, etidronate and zoledronic acid. Six studies reported data on BMD, seven on BTMs, and 13 on fractures. As such, this is the largest systematic review to collectively examine data on BP drug holidays. Fourteen real‐world studies were published since the release of American Society for Bone and Mineral Research (ASBMR) Taskforce Guidelines on Long‐Term BP Treatment in 2016, providing a multifaceted and updated perspective on the issue since these guidelines.

## Perspectives From Clinical Trials

### BMD and BTMs

Clinical trials have examined the effects of drug holidays predominantly in postmenopausal women, with a wide range of treatment durations from 1 to 7 years, and holidays ranging 7 months to 5 years. See Table 1 for a full list of studies.

**Table 1:** Randomized Controlled Trials Related to BP Drug Holidays

Author	Year	Size	Patient population	Methodology	Medication	Treatment duration	Discontinuation duration	Outcome measure	Outcome
Black^27^	2012	ZOL6 = 616 ZOL3PLC3 = 617	Postmenopausal women between 65 and 89 years with FN T‐score ≤ −2.5 with or without existing VF, or T‐score ≤ −1.5 with at least 2 mild VFs or one moderate fracture.	Extension of RCT	Zoledronic acid	3 years	3 years	BMD, rate of fractures， BTM	ZOL3PLC3‐ lower LS and DR BMD at 4.5 years, lower FN, TH, LS BMD at 6 years Higher rate of morphometric VF, no difference in nonvertebral/hip or clinical VF. Absolute difference of 47% in P1NP change at 4.5 year mark but reduced to 14% by 6 years.P1NP still remained below baseline after treatment discontinuation.
Cosman^40^	2014	ZOL6 = 616 ZOL3PLC3 = 617	Postmenopausal women between 65 and 89 years with FN T‐score ≤ −2.5 with or without existing VF, or T‐score ≤ −1.5 with at least 2 mild VFs or one moderate fracture.	Extension of RCT	Zoledronic acid	3 years	3 years	Predictors of VFs	ZOL3PLC3‐ RF for new VF include low TH or FN BMD ≤‐2.5 at 3 years. Higher rate of new VF at end of follow‐up in ZOL3PLC3 versus ZOL6 among women without VF in first 3 years. In ZOL3PLC3 with hip T‐score >−2.5 with no incident fracture and <1 risk factor, risk for subsequent fracture over next 3 years is low.
Black^28^	2015	ZOL9 = 95 ZOL6PLC3 = 95	Postmenopausal women between 65 and 89 years with FN T‐score ≤ −2.5 with or without existing VF, or T‐score ≤ −1.5 with at least 2 mild VFs or one moderate fracture.	Extension of RCT	Zoledronic acid	6 years	3 years	BMD, rate of fractures, BTM	ZOL6PLC3‐ lower TH BMD at year 8. No difference between groups for TH or FN BMD at year 9. No difference between groups for VF or clinical fracture rates at year 9. P1NP, CTX, BSALP in normal range in both groups. ZOL6PLC3‐ P1NP higher at year 7 and BSALP higher at year 9
Watts^38^	2008	RIS3OFF1 = 309 PLC4 = 290	>5 year postmenopausal women <85 years with either 2 or more VFs, or 1 VF with low LS BMD T‐score < −2	Extension of RCT	Risedronate	3 years	1 year	BMD, rate of fractures, BTM	RIS3OFF1‐ LS, FN, TRO BMD all reduced in year off treatment but higher than baseline and PLC. Risk of VF lower in RIS3OFF1 than PLC. Urine NTX increased off treatment, lower than baseline but similar to PLC. BSALP increased off treatment, similar to baseline and PLC.
Eastell^39^	2011	RIS7OFF1 = 31 PLC5RIS2OFF1 = 30	>5 year postmenopausal women <85 years, with at least 2 VFs	Extension of RCT	Risedronate	7 years	1 year	BMD, BTM	RIS7OFF1 and PLC5RIS2OFF1‐ TH and TRO BMD decreased but LS and FN BMD maintained/increased from year 7 to year 8. In both groups, NTX/Cr increased in the year off‐treatment towards baseline.
Black^29^	2006	ALN10 = 662 ALN5PLC5 = 437	Postmenopausal women between 55 ad 81 years with FN BMD <0.68/ T‐score < −1.6	Extension of RCT	Alendronate	5 years	5 years	BMD, rate of fractures, BTM	ALN5PLC5‐ decrease in TH, FN, TRO, LS, FOR BMD, higher rate of clinical VF but no difference in morphometric VF. CTX, BSALP increased but lower than baseline
Schwartz^41^	2010	ALN10 = 662 ALN5PLC5 = 437	Postmenopausal women between 55 and 81 years with FN BMD <0.68/ T‐score < −1.6	Extension of RCT	Alendronate	5 years	5 years	Predictors of VFs	In women without VF at 5 years and FN T‐score < −2.5, ALN10 reduced non‐VF.
Bauer^31^	2014	ALN10 = 662 ALN5PLC5 = 437	Postmenopausal women between 55 and 81 years with FN BMD <0.68/ T‐score < −1.6	Extension of RCT	Alendronate	5 years	5 years	Predictors of VFs	ALN5PLC5–22% sustained ≥1 clinical fracture. Predictors of fracture‐ older age, lower TH/FN BMD at 5 years. 1 year change in hip BMD, BTM not predictive.
Ensrud^30^	2004	ALN10 = 662	Postmenopausal women between 55 and 81 years with FN BMD <0.68/ T‐score < −1.6	Extension of RCT (interim analysis)	Alendronate	5 years	3 years	BMD, BTM	ALN5PLC3 decline in TH, FN, TRO, TB, forearm but increase at LS, and higher than baseline. Increase in NTX and BSALP.
		ALN5PLC5 = 437							
Torino^35^	2000	ALN7 = 235	Postmenopausal women with LS BMD ≤‐2.5	Extension of RCT	Alendronate	5 years	2 years	BMD, BTM	ALN5PLC2‐ no decline in LS/hip BMD, small but significant decline in FOR, total body BMD Small increase in urine NTX, BSALP after year 5
		ALN5PLC2 = 115							
Bone^36^	2004	ALN10 = 164	Postmenopausal women with LS BMD ≤‐2.5	Extension of RCT	Alendronate	5 years	5 years	BMD, rate of fractures, BTM	ALN5PLC5‐ significant decrease in FN, TH, FOR BMD, no change in LS, TRO BMD. Urine NTX, BSALP increased within 1 year but below baseline No difference in morphometric VFs
		ALN5PAC5 = 83							
Stock^33^	1997	n = 188 (total) PLC2OFF1 ALN(5 mg)2OFF1 ALN(10 mg)2OFF1 ALN(20 mg)1PLC1OFF1 ALN(40 mg)PLC1OFF1	>5 year postmenopausal women between 42 and 75 years with LS BMD ≤‐2	Extension of RCT	Alendronate	1 to 2 years	1 to 2 years	BMD, BTM	ALN(5 mg)2OFF1‐ decrease in LS BMD at 3 years ALN(10 mg)2OFF1‐ decrease in TH BMD at 3 years ALN(20 mg)1PLC1OFF1‐ no change ALN(40 mg)1PLC1OFF1‐ no change uDPD, NTX, BSALP, OC increased off‐treatment but less than PLC or baseline.
Ravn^37^	2000	ALN(5 mg)5 = 52	6‐36mo postmenopausal women between 40 and 59 years with LS BMD between −2 and + 2.	RCT	Alendronate	2 years	3 years	BMD, BTM	ALN(20 mg)2PLC1OFF2‐ bone loss varied from 1.8% to 7.5% across various sites. LS, TRO BMD increased 2.5% to 2.8% at end of 5 years but not different from baseline at FN, TB
		PLC3ALN2 = 56							
		ALN(20 mg)2PLC1OFF2 = 52							
									Urine NTX, CTX increased but remained 40% to 60% below baseline at 5 years.
Wasnich^34^	2004	PLC6 = 132	Postmenopausal women between 45 and 59 years.	RCT	Alendronate Estrogen/progestin	2 to 4 years	2 to 4 years	BMD, BTM	ALN2PLC4, ALN4PLC2‐ BMD decreased at all sites off‐treatment, but higher than baseline.
		ALN6 = 90							
		ALN4PLC2 = 86							
		ALN2PLC4 = 94							
Miller^32^	2008	ALN2OFF2 = 47	Postmenopausal women <80 years with LS T‐score − 1.8 to −4.0 or FN/TH T‐score − 1.8 to −3.5	RCT	Denosumab, Alendronate	2 years	1 to 2 years	BMD, BTM	ALN2OFF2‐ small decrease in LS BMD, higher decreases at TH, DR. Increase in CTX, BSALP but below baseline

AHR = adjusted hazard ratio; ALN = alendronate; BSALP = bone‐specific alkaline phosphatase; CI = confidence interval; CTX = C‐terminal telopeptide of type 1 collagen; DMAB = denosumab; DR = distal radius; FN = femoral neck; FOR = forearm; HR = hazard ratio; IV = intravenous; LS = lumbar spine; MOF = major osteoporotic fracture; NTX = N‐terminal telopeptide of type 1 collagen; OC = osteocalcin; OR = odds ratio; P1NP = procollagen type 1 N propeptide; PLC = placebo; PY = patient‐years; RF = risk factor.RIS = risedronate; RR = relative risk; TB = total body; TH = total hip; TRO = trochanter; uDPD = urinary deoxypyridinoline; VF = vertebral fracture; ZOL = zoledronic acid; ZOL3OFF1 = zoledronic acid for 3 years; off‐treatment for 1 year.

In the Health Outcomes and Reduced Incidence with Zoledronic Acid Once Yearly–Pivotal Fracture Trial (HORIZON‐PFT) trial, subjects with postmenopausal osteoporosis were randomized to continue annual zoledronic acid for a total of 6 years or discontinue after 3 years. Significant differences in BMD between the groups were demonstrated at 4.5 years and were more pronounced at 6 years.^(^ ^27^ ^)^ Persistent treatment with zoledronic acid from 3 to 6 years resulted in a further 3.2% increase in lumbar spine bone density compared to a 1.18% increase from 3 to 6 years in those discontinuing treatment at 3 years. Bone density remained stable in the hip in those on persistent treatment, while a decline of 1.58% in those undertaking a drug holiday after 3 years was observed.^(^ ^27^ ^)^ The decline in BMD was most significant after 3 years of drug holiday. The relatively small percentage change in BMD was associated with reduced incidence of vertebral but not nonvertebral fractures between the groups.^(^ ^27^ ^)^

BTMs differed significantly between the groups with a reduction in procollagen type 1 N propeptide (P1NP) of 23% in the persistent treatment group and a 24% increase in the drug holiday group at year 6.^(^ ^27^ ^)^ No further gains in BMD were achieved in a further extension study to 9 years compared to 6 years.^(^ ^28^ ^)^ P1NP and C‐terminal telopeptide of type 1 collagen (CTX) increased only slightly from year 6 to 9 in the discontinuation group, while the increase in bone‐specific alkaline phosphatase (BSALP) was more noticeable at the 9‐year mark, all remaining significantly reduced compared to pretreatment levels, indicating a slow offset of antiresorptive efficacy after 6 years of treatment.^(^ ^28^ ^)^

In the Fracture Intervention Trial Long‐term Extension (FLEX) study, patients receiving alendronate 70 mg weekly for 5 years showed a decline in BMD during the 5 years off treatment, with total hip BMD declining 3.38% and essentially returning to pretreatment levels, while the persistent treatment group saw a decline of 1.02% with ongoing treatment.^(^ ^29^ ^)^ BMD loss at the hip was significant within 3 years of drug holiday initiation, suggesting consideration of BP resumption at this time point.^(^ ^30^ ^)^ Changes in lumbar spine BMD were difficult to interpret due to age‐related degenerative artifact, with an increase by 1.52% during 5 years off treatment (and 10.99% from baseline). Persistent treatment from 5 to 10 years conferred a further 5.26% increase in lumbar spine.^(^ ^29^ ^)^

Three years into the alendronate holiday in the FLEX study, BSALP and N‐terminal telopeptide of type 1 collagen (NTX) increased by 15.4% and 21.6%, respectively, while they remained suppressed in the persistent treatment groups.^(^ ^30^ ^)^ The greatest increase in BSALP and NTX occurred by the first year off‐treatment, and remained stable by the 3‐year time‐point. After 5 years of an alendronate holiday, there was >50% higher serum CTX and NTX, and 28.1% higher BSALP compared to those who received alendronate for 10 years, although CTX and BSALP were 7% lower and NTX 24% lower than baseline.^(^ ^29^ ^)^ BTMs in a post hoc analysis were not predictive of fractures during a BP drug holiday, with neither the tertile with the greatest increase in NTX and BSALP, nor those demonstrating a ≥30% increase in NTX and BSALP at 1 or 3 years posttreatment cessation, predicting an increased risk of fracture.^(^ ^31^ ^)^ However, as fractures were infrequent in this extension arm of the FLEX study, and with wide confidence intervals for BTMs, study power to detect a predictive correlation between BTM trends and resurgent fractures in the drug holiday group was inadequate.

Shorter treatment duration with 2 years of alendronate 70 mg weekly followed by 2 years off treatment showed small decreases at the lumbar spine, but larger decreases at the hip and distal radius.^(^ ^32^ ^)^ Studies examining drug holidays following daily dosing regimens of alendronate have been conducted. Following 2 years of alendronate 5 or 10 mg daily, subjects experienced significant, early declines in bone density after a 12‐month holiday, indicating that drug frequency (ie, daily or weekly) does not influence offset of anti‐resorptive effect.^(^ ^33^ ^)^ Subjects receiving 4 years of daily alendronate 5 mg daily followed by a 2‐year holiday had significantly greater gains in bone density at study end than those receiving the converse (ie, 2 years of alendronate with 4 years off treatment).^(^ ^34^ ^)^

Despite the use of higher doses of alendronate than currently used in clinical practice, consistent declines in femoral neck and total hip bone density during 2‐year to 5‐year drug holidays were reported (ie, dose regimens 20 mg daily for 2 years followed by 5 mg daily for 3 years).^(^ ^35^, ^36^ ^)^ Similarly, daily doses of alendronate of 20 mg for 2 years followed by 3 years off treatment led to a return in femoral neck BMD to baseline at study concludion.^(^ ^37^ ^)^ Alendronate treatment durations between 1 and 5 years with off‐treatment durations of 1 to 4 years all resulted in increases in both formation and resorptive markers, but these remained below pretreatment levels.^(^ ^32^, ^33^, ^34^, ^37^, ^38^ ^)^

Drug holidays in risedronate users resulted in earlier declines in bone density than those receiving zoledronic acid or alendronate. In one study, in the first year of drug holiday following 3 years of risedronate 35 mg weekly, lumbar spine BMD declined by 0.83% and total hip by 1.57%, but these values remained higher than baseline and the placebo group (treatment‐difference 2.60% and 3.08%, respectively).^(^ ^38^ ^)^ Urine NTX increased significantly off treatment, similar to levels in placebo subjects, but still significantly lower than baseline, while increase in BSALP had returned to baseline within the 1‐year holiday.^(^ ^38^ ^)^ Despite treatment with risedronate for up to 7 years, significant declines following a 1‐year holiday at total hip were apparent.^(^ ^39^ ^)^ Urine NTX/Cr levels increased significantly in the 1 year off treatment regardless of treatment for 2 or 7 years, but remained below baseline.^(^ ^39^ ^)^

### Fractures during BP holiday

Subjects embarking on a drug holiday after 3 years of annual zoledronic acid had double the risk of morphometric vertebral fracture compared to those persisting for a further 3 years (6.2% versus 3%, respectively).^(^ ^27^ ^)^ However, anti‐fracture effects in all clinical fractures including clinical vertebral and hip fractures were retained by 3 years of treatment. The greatest risk factor for a morphometric vertebral fractures in those discontinuing zoledronic acid after 3 years was a morphometric vertebral fracture while on‐treatment, imparting a 4.8‐fold increase in risk (95% confidence interval [CI], 1.4–16.8).^(^ ^40^ ^)^ Second, either femoral neck or total hip *T*‐score ≤ −2.5 standard deviation (SD) at end of treatment was an important predictor of off‐treatment morphometric vertebral fractures.^(^ ^40^ ^)^ For every *T*‐score SD decline in femoral neck/total hip BMD, the odds of a new morphometric vertebral fracture increased threefold (95% CI, 1.5–6.0).^(^ ^40^ ^)^ Patients with these risk factors may therefore benefit from ongoing treatment to 6 years.

Separately, nonvertebral fractures in subjects on a drug holiday were predicted by the occurrence of nonvertebral fractures on‐treatment (hazard ratio [HR] 2.5; 95% CI, 1.2–5.3), prevalent vertebral fracture (HR 3.0; 95% CI, 1.4–6.3), and low total hip *T*‐score at end of treatment (for every 1 SD decline, HR 1.7; 95% CI, 1.2–2.6).^(^ ^40^ ^)^ Higher‐risk patients should therefore continue treatment with zoledronic acid to 6 years. Treatment from 6 to 9 years in contrast conferred no further benefits in morphometric vertebral or clinical fracture reduction.^(^ ^28^ ^)^

Among subjects in the FLEX trial, those who received alendronate for 5 years compared to 10 years, demonstrated no difference in cumulative risk of nonvertebral fractures (risk ratio [RR] 1.00; 95% CI, 0.76–1.32).^(^ ^29^ ^)^ Subjects on a drug holiday were, however, twice as likely to experience clinical vertebral fractures in the holiday period (5.3% versus 2.4%), but no difference in morphometric vertebral fractures was seen.^(^ ^29^ ^)^ Predictors of holiday‐related fractures included lower femoral neck or total hip BMD with the highest risk in those in the lowest tertile (femoral neck BMD −4.1 to −2.5 SD, total hip BMD −4.2 to −2.3 SD) and older age (mean age 76.2 versus 73.1).^(^ ^31^ ^)^ Lower pretreatment BMD or prevalent vertebral fracture was associated with a greater risk of holiday‐related fracture, but did not reach statistical significance.^(^ ^29^ ^)^ Femoral neck *T*‐score ≤ −2.5 SD at the end of 5 years of alendronate treatment, independent of fracture history, conferred a higher rate of nonvertebral fracture in those undertaking a holiday at 5 years compared to those on 10 years of treatment (RR 0.50; 95% CI, 0.26–0.96).^(^ ^41^ ^)^

In contrast, in the Alendronate Phase III Osteoporosis Treatment Study, there was no difference in rate of morphometric vertebral fractures among women who were treated with 10 years of 5 mg alendronate daily (13.9%), 10 years of 10 mg alendronate daily (5.0%), or discontinued treatment after 5 years (6.6%).^(^ ^36^ ^)^ Possible reasons for this was the considerably younger age of subjects in this study than the FLEX cohort (63 versus 73 years) and differences in baseline fracture prevalence.^(^ ^36^ ^)^

Three years of risedronate maintained anti‐fracture effects during a 1‐year drug holiday with only 6.5% of subjects sustaining a vertebral fracture compared to 11.6% of those never receiving treatment.^(^ ^38^ ^)^ However clinical trials have not directly compared fracture rates in persistent risedronate users compared to those undertaking a holiday.

### Conclusions from clinical trials

Evidence from RCTs indicate that BP drug holidays led to declines in BMD across various skeletal sites, although final BMD was typically above baseline. Although effects of annual zoledronic acid persisted beyond 6 years, earlier BMD declines in patients previously treated with alendronate and risedronate were seen, most notably in risedronate users who demonstrated BMD loss as early as 1 year off treatment.^(^ ^38^, ^39^ ^)^ BTMs increased soon after treatment cessation, although in most studies remained significantly below pretreatment levels at the end of follow‐up, indicating durability of BP effect on bone turnover. There was an increased risk of vertebral and nonvertebral fractures during drug holidays in certain high‐risk groups. In both zoledronic acid and alendronate studies, predictors for fracture during drug holidays include older age, lower hip BMD (*T*‐score ≤ −2.5) at the end of BP treatment and incident fracture during treatment—these patients benefited from ongoing treatment. Thus, in the absence of these significant risk factors, drug holidays could be considered in subjects after prolonged treatment, after 6 years of zoledronic acid, or 5 years of alendronate, whereas risedronate users should be followed closely due to a more rapid offset of anti‐resorptive effect.

## Perspectives From Real World Studies

An emerging number of real world studies have shed light on outcomes of BP drug holidays from everyday clinical practice and present another perspective on this issue (Table 2). Owing to inherent differences in study design, patient populations, methods of analysis, and outcome measures in these studies, direct comparison between real‐world studies poses challenges. Notwithstanding, this section aims to extrapolate key learning points from real‐world studies, offering new insights beyond the rigid framework of clinical trials.

**Table 2:** Real‐World Studies Related to BP Drug Holidays

Author	Year	Size	Patient population	Methodology	Medication	Treatment duration	Discontinuation duration	Outcome measure	Outcome
Bagger^55^	2003	203	Postmenopausal women	Prospective observational study post RCT	ALN	2, 4, 6 years	3, 5, 7 years	BMD, CTX, OC	Rate of bone loss during BP holiday similar to normal postmenopausal bone loss. BTMs increased however remained lower than PLC. Significant sustained suppression of BTM in longest treatment group of 6 years.
Curtis^43^	2008	9,063	Females aged 60 to 78 years.	Population based cohort study	ALN (77%), RIS (23%)	≥2 years	0 to 6 months, 6 to 12 months, >12 months	Hip fracture	No significant risk of hip fracture in patients with high compliance at 2 years (MPR ≥80%) or compliant for 3 years.
Gallagher^47^	2008	44,531	Females (36,164) and males (8,367) >18 years	Population study	ALN (74.1%), RIS (25.9%)	0 to 6 months, 6 to 24 months, >24 months	0 to 6 months, >6 months	Fracture risk	Lower hip fracture risk by 22% in current BP users versus BP holiday group. Increased fracture risk in patients with low compliance.
Chiha^46^	2013	209	Female (191) and male (18) with osteoporosis or osteopaenia	Retrospective chart review	ALN (67%), RIS (27.3%), Ibandronate (5.7%)	Mean 6.7 ± 2.7 years	2 to 4 years	Fracture, BMD, ALP, CTX	5.2% developed a fracture within 2 years of BP holiday. RF included lower BMD and older age. No significant changes in LS BMD at 4 years; significant decline in FN BMD at 2 years.
Roberts^42^	2016	134	Female (121) and male (13) with osteoporosis	Retrospective study	ALN (57%), RIS (15%), IV BPs (28%)	5.9 years	12 to 18 months, 24 to 30 months	BMD, CTX, P1NP and FRAX	BMD decreased significantly at all sites after 12 to 18 months of BP holiday. CTX and P1NP increased significantly after 12 to 18 months of BP holiday. High FRAX score > 25% predicts bone loss at FN.
Xu^49^	2016	208	Female (87.5%), patients with osteoporosis	Single centre retrospective cohort	ALN (71%), RIS (29%)	≥2 years, Average 5.2 ± 2.3 years	3.3 ± 1.7 years	BMD	TH BMD declined significantly within 1 year of BP holiday, especially in lean patients. LS and FN BMD remained stable within 2 years of BP holiday. Greater BMD decline in former RIS compared to ALN users.
Curtis^58^	2017	156,236	Women on long term BP (62,676 stopped BP)	Population based cohort	ALN (71.7%), ZOL (16.2%)	≥3 years	Up to 3 years	Hip fracture	Hip fracture rates lowest among current BP users. Hip fracture risk increased with duration of BP holiday, up to 39% after 2 years.
Mignot^59^	2017	183	Postmenopausal women with OP	Retrospective analysis	ALN (44.2%), RIS (39.9%), ZOL (10.9%), Ibandronate (4.9%)	Oral BP 5 years or IV BP 3 years	6 to 36 months	Clinical fracture	Higher fracture risk in BP holiday group (16.1%) versus continued group (11.9%). New fracture during drug holiday HR = 1.40.
Liel^53^	2017	211	Females with osteoporosis	Retrospective	ALN (86.3%),RIS (13.7%)	Mean 7.2 ± 3.1 years	Median 1.25 years	urine DPD	uDPD increased significantly after 1 year of BP holiday.
Adams^44^	2018	39502	Women ≥45 years and on ≥3 years of BP with ≥50% adherence.	Population based retrospective cohort	ALN (98.8%), RIS, ZOL, Ibandronate, Etidronate, Pamidronate,	≥3 years	4.1 ± 2.4 years for non persistent users, 4.9 ± 2.5 years for BP holiday	OP related fracture	No increase in risk of MOF, hip fracture, or VFs in BP holiday group. After risk stratification, BP holiday group still has lower fracture risks versus persistent and non‐persistent groups (HR 0.88; 95% CI, 0.78 to 0.99 and HR 0.70; 95% CI, 0.61 to 0.81; respectively)
Bindon^48^	2018	401	Female (371) and male (30) with osteoporosis or osteopaenia	Retrospective chart review	ALN (61.6%), RIS (34.4%), Ibandronate (13.3%), ZOL (6.91%)	Average 6.34 ± 3.23 years	6 months to 6 years	Fracture	15.4% of patients developed a fracture during BP holiday. Fracture incidence increases with duration of BP holiday.
Curtis^57^	2020	81,427 (28% on BP holiday)	Females ≥65 years.	Observational cohort study Medicare data	ALN (72.8%), RIS (8.4%), ZOL (9.8%), Ibandronate (9%)	≥3 years	≥2 years	Hip/humeral/clinical vertebral fracture	In the ALN cohort, increased risk in hip fracture (aHR =1.3, 1.1 to 1.4), humerus fracture (aHR =1.3, 1.1 to 1.66) and clinical VF (aHR =1.2, 1.1 to 1.4) with BP holiday >2 years. Result similar for RIS. No significant fracture risk in ZOL and ibandronate.
Pfeilschifter^50^	2020	1,973	Men aged ≥59 years and postmenopausal women	Prospective observational cohort	ALN (61.53%), RIS (33.91%), ZOL (11.76%), Ibandronate (25.14%)	≥4 years	Upto 25 months	Fracture risk and mortality	No significant risk in fracture and mortality in BP holiday group. Prevalent VF increases the risk of MOF in a longer BP holiday.
Aboughanima^54^	2020	85	Postmenopausal women	Single center prospective study	ALN (96.5%), ZOL (3.5%)	5 years ALN or 3 years ZOL with ≥80% adherence	24 months	VF, BMD, CTX, ALP	RF for VF included older age and history of previous fracture. Significant increase in CTX. Significant decline in FN and TH BMD in fracture group only.
Statham^51^	2020	158	131 women and 27 men, with a mean age of 71 years	Retrospective analysis	ALN (59%), RIS (33%), Ibandronate (6%), ZOL (2%)	Mean 8 years	4 and 12 months	CTX	32% of patients showed inadequate suppression of CTX at baseline. CTX increased by 28% at 4 months and 53% by 12 months for >25% of patients.
Sølling^52^	2021	136	Postmenopausal women and men >50 years.	Cohort study	ALN	≥5 years	24 months	BMD, CTX, P1NP, osteocalcin	BMD decreased significantly and BTMs increased within the reference range over 2 years of BP holiday. Increase in CTX after 3 months is associated with greater bone loss at the hip at 12 and 24 months.
Sølling^45^	2021	55,369	Women >52 years and men >50 years. 1865 ALN discontinuers and 29619 ALN continuers.	Population based cohort study	ALN	> 5 years	Up to 5 years	Fracture	No increased fracture risk in BP holiday group after 5 years of BP use. Old age (>80 years) was the strongest determinant for fracture during BP holiday.
Fu^56^	2021	3,663	Patients ≥50 years.	Population based cohort study	DMAB, ALN (57.6%), RIS (0.1%), ZOL (29.6%), Ibandronte (12.7%)	1 to 2 years	12 months	Major osteoporotic fracture	Increased MOFs and VFs in non‐persistent BP users after 2 years of BP treatment.
Hayes^60^	2022	50,154	Patients ≥66 years, 82% female	Population based study;	ALN, RIS	≥3 years	Up to 3 years	Hip fracture	Higher hip fracture rates among RIS than ALN users (12.4 and 10.6 per 1000 PY; HR 1.18 [95% CI, 1.04 to 1.34]). Higher fracture risk in men (HR 1.37 [95% CI 0.95 to 1.97]) versus women (HR 1.15 [95% CI 1.01 to 1.33]) in RIS users. Increased fracture risk with RIS began at 2 years into BP holiday.

AHR = adjusted hazard ratio; ALN = alendronate; BSALP = bone‐specific alkaline phosphatase; CI = confidence interval; CTX = C‐terminal telopeptide of type 1 collagen; DMAB = denosumab; DR = distal radius; FN = femoral neck; FOR = forearm; HR = hazard ratio; IV = intravenous; LS = lumbar spine; MOF = major osteoporotic fracture; NTX = N‐terminal telopeptide of type 1 collagen; OC = osteocalcin; OR = odds ratio; P1NP = procollagen type 1 N propeptide; PLC = placebo; PY = patient‐years; RF = risk factor.RIS = risedronate; RR = relative risk; TB = total body; TH = total hip; TRO = trochanter; uDPD = urinary deoxypyridinoline; VF = vertebral fracture; ZOL = zoledronic acid; ZOL3OFF1 = zoledronic acid for 3 years; off‐treatment for 1 year.

We reviewed 19 real‐world studies, including population‐based studies, single‐center cohort studies, and observational studies post‐RCTs that evaluated BP holiday‐related effects on BMD, BTMs, and fractures. One study also examined the utility of the Fracture Risk Assessment Tool (FRAX) score in predicting BMD and the implication on fracture outcomes during drug holiday.^(^ ^42^ ^)^ In general, fracture events were sourced from electronic medical records, patient registries including healthcare databases, hospital admission, and emergency presentation codes.^(^ ^43^, ^44^, ^45^ ^)^ Medication adherence, measured using medication possession ratios (MPRs) or proportion of days covered (PDC), were reported with the caveat that MPR may overestimate adherence when patients frequently refill prescriptions. Studies circumvented the issue of confounding by adjusting for clinical risk factors that could impact on drug holiday decisions in these studies, propensity score matching for control cohorts, and use of multivariate models.^(^ ^43^, ^44^, ^45^ ^)^

Postmenopausal women with low BMD were the predominant group examined in these studies. Nine studies included men who represented 10% to 20% of the cohort, and premenopausal women were examined in one study.^(^ ^42^, ^45^, ^46^, ^47^, ^48^, ^49^, ^50^, ^51^, ^52^ ^)^ The main BP used was alendronate, followed by risedronate, zoledronic acid, and ibandronate.

### BMD and BTMs

Consistent with the findings from clinical trials, real‐world studies demonstrated early increases in bone turnover, coupled with significant declines in bone density as early as 1 to 2 years following the initiation of a drug holiday.^(^ ^42^, ^52^, ^53^, ^54^ ^)^ In a cohort from Denmark, a significant reduction in total hip BMD of 2.65% associated with an increase in serum CTX by 60% occurred during a 2‐year drug holiday in subjects who had previously received long‐term alendronate therapy (median of 7 years).^(^ ^52^ ^)^ Significant increases in P1NP and CTX after 3 months of drug holiday independently predicted greater bone density loss at 12 and 24 months.^(^ ^52^ ^)^ However, in a UK cohort, BTMs did not predict bone density loss in females undertaking a drug holiday.^(^ ^42^ ^)^ Rather, lower baseline body mass index (BMI), weight loss during BP holiday, and a high FRAX score (>25% risk of major osteoporotic fractures over 10 years) were independent predictors of declines in total hip and femoral neck BMD.^(^ ^42^, ^49^ ^)^

Lumbar spine changes were inconsistent across the studies, possibly relating to varying degrees of degenerative artifact over different study periods. Although shorter drug holidays led to significant declines in lumbar spine BMD,^(^ ^42^ ^)^ changes were confounded by degenerative artifact in holidays beyond 2 years and lumbar BMD was no longer an accurate marker of long‐term fracture risk.^(^ ^45^, ^46^, ^49^, ^54^ ^)^

Drug holidays led to a more rapid decline in bone density and increases in bone turnover in subjects previously prescribed risedronate versus alendronate.^(^ ^49^, ^53^ ^)^ In a study examining different durations of alendronate use in postmenopausal women, drug holidays did not lead to significant changes in bone density.^(^ ^55^ ^)^ Subjects in this study, however, had a lower risk of fracture, were in the younger postmenopausal category (40–59 years), with baseline BMD in the normal or osteopenic range, and without prior minimal‐trauma fracture. Over drug holiday periods of 3 to 7 years, bone turnover gradually returned to background rates of postmenopausal bone turnover, but CTX and P1NP remained significantly lower than the control group.

### Fractures during BP drug holiday

Twelve studies evaluated the incidence of fractures during a BP drug holiday. Three population‐based studies from the United States and Europe with sample sizes as high as 50,000 subjects did not find significant increase in fractures in patients discontinuing BP after a period of use of more than 3 years and monitored for up to 5 years.^(^ ^44^, ^45^, ^50^ ^)^ These included population‐based studies with fracture outcomes extracted from healthcare databases^(^ ^44^, ^45^ ^)^ and an interview‐based prospective observational study with fracture self‐reporting.^(^ ^50^ ^)^ It is possible that a proportion of fractures were not accounted for in these real‐world studies. Predominantly, postmenopausal women with a mean age of 69 to 75 years with adherence rates of >50% to 80%, prescribed any of a number of BPs including oral and intravenous formulations, were included.^(^ ^44^, ^45^, ^50^ ^)^

A large retrospective study of a racially and socioeconomically diverse cohort from the United States identified adherence as the main determinant of sustained reductions in fractures during a drug holiday.^(^ ^44^ ^)^ Subjects in the BP holiday group who had greater adherence to oral BPs (MPR > 50%) while on treatment had a 30% reduction compared to nonpersistent BP users, defined by MPR < 50% or no BP use for <12 months, in the 3‐year‐long drug holiday.^(^ ^44^ ^)^ Similarly, another study of postmenopausal women between 60 and 78 years reported no increase in hip fracture in a 12‐month drug holiday in patients highly adherent to oral BP for 2 years.^(^ ^43^ ^)^ The drug holiday group had lower baseline FRAX score, higher BMD, and few prior fractures, suggesting at baseline these patients had lower risk of fracture and hence were considered suitable candidates for a drug holiday. However, even after adjustment for these clinical factors that influence drug holiday decisions, fracture rate remained consistently low in the BP holiday group.

A nationwide population‐based cohort from Denmark reported similar fracture rates in patients undertaking a 5‐year drug holiday or persisting with treatment after at least 5 years of alendronate use.^(^ ^45^ ^)^ Importantly, both men and postmenopausal women were included in this study, factors impacting treatment decisions were adjusted using Cox proportional hazards regression analysis and high adherence rates while on treatment were reported, likely protective against holiday fractures.

In a large US healthcare database, patients embarking on a 12‐month drug holiday were protected from hip fracture if they were adherent with oral BPs for at least 2 years.^(^ ^43^ ^)^ A nationwide cohort study from Taiwan compared fracture rates in patients discontinuing denosumab versus bisphosphonates. Although fracture rates increased significantly in patients discontinuing denosumab versus persistent users, BP drug holidays were not associated with fractures particularly in the subgroup with high adherence during the period of use.^(^ ^56^ ^)^ Conversely, patients nonadherent to oral BPs displayed a higher risk of fractures in the drug holiday period.^(^ ^43^ ^)^

Apart from poor adherence, additional risk factors for holiday‐related fractures included lower baseline BMD, previous fractures, and age >78 years compared to 50 to 60 years.^(^ ^45^, ^57^, ^58^ ^)^ Three additional studies reported a similar trend in age as a risk factor.^(^ ^46^, ^48^, ^54^ ^)^ In one study, every one unit decrease in the femoral neck *T*‐score at baseline raised the odds of a new fracture by 37% during a drug holiday over 6 years.^(^ ^48^ ^)^ Prevalent fracture was a significant predictor of vertebral fracture during drug holidays^(^ ^54^ ^)^ imparting a 3.53‐fold increase in risk of major osteoporotic fracture in a German study, most pronounced in those with holidays >12 months.^(^ ^50^ ^)^

In several studies, fractures were detected soon after the onset of a drug holiday, as early as 6 months.^(^ ^46^, ^48^, ^59^ ^)^ In a large study of over 80,000 postmenopausal women >65 years, there was a trend for more holiday‐related fractures in subjects discontinuing alendronate and risedronate versus zoledronic acid and ibandronate.^(^ ^57^ ^)^ A smaller retrospective study reported a slightly higher incidence of holiday‐related fractures in patients discontinuing oral versus intravenous BPs.^(^ ^48^ ^)^ Risedronate users had a relative 18% higher risk of hip fractures compared to alendronate users during a 3‐year‐drug holiday.^(^ ^60^ ^)^

### Conclusion from real‐world studies

These studies provide a complementary picture on the relative safety of drug holidays in the real‐world setting, in which deliberate assessment of patient fracture risk and holiday timing would be undertaken. Even after adjusting for a variety of factors impacting on these decisions, drug holidays remained safe after a period of BP use >3 years, with the exception of patients displaying particular risk factors.^(^ ^44^, ^45^, ^50^ ^)^ These included older age, lower baseline BMD, and previous fracture, all consistent with those identified in clinical trials. Low adherence was a consistent risk factor for holiday‐related fractures, identified in this real‐world database. Changes in BMD and bone turnover were more pronounced in users of oral BPs versus intravenous BPs during drug holidays, with the suggestion for more frequent fractures in former group. A shorter drug holiday may therefore be considered for users or oral BPs, in particularly risedronate, as the anti‐fracture effect is likely less durable.

## Discussion

Current guidelines on long‐term BP treatment and drug holidays rely on the individualisation of a patient's fracture risk. The National Osteoporosis Foundation (NOF) recommends a comprehensive risk assessment after 3 to 5 years of BP treatment, with particular consideration of intercurrent fracture history, new chronic diseases or medications, height measurement, BMD testing, and vertebral imaging.^(^ ^61^ ^)^ Similarly, the ASBMR recommends a drug holiday after 5 years of oral BP or 3 years of intravenous BP in postmenopausal patients with low facture risk and hip *T*‐score > −2.5 SD.^(^ ^62^ ^)^ A meta‐analysis on the effect of BP drug holidays supports the safety of this practice in women without low hip BMD after 3 to 5 years of treatment, whereas those with low hip BMD would benefit from persistent treatment.^(^ ^63^ ^)^ Guidelines by the International Osteoporosis Foundation (IOF) warned of increased fracture risk following the cessation of antiresorptive therapy, especially in those with advanced age and low BMD.^(^ ^64^ ^)^ These guidelines, however, concede knowledge gaps in long‐term treatment regimens, the clinical utility of surrogate markers of fracture risk, and the ideal duration for a drug holiday. Bisphosphonate‐specific comparisons, with data on patients undergoing holidays from risedronate, currently the mostly commonly prescribed oral BP,^(^ ^60^ ^)^ were also lacking. Since the publication of these guidelines, an increasing number of real‐world studies have shed new light on these questions.

We conducted the largest systematic review on this topic with 15 RCTs and 19 real‐world studies examining the impact of BP drug holidays on bone turnover, bone density, and fractures. A summary of key points raised from these studies is found in Table 3.

**Table 3:** Key Considerations for Bisphosphonate Drug Holidays

Drug holidays should not be a universal practice in all long‐term BP users. High‐risk patients who embark on an injudicious BP drug holiday are at risk of fragility fractures.
In the appropriate setting, BP drug holidays are safe, reduce the risk of AFF while benefiting from continued anti‐fracture effects of long‐term BPs. Drug holidays neutralize the risk of AFF after long‐term treatment. Beyond two years, the increased risk of fragility fracture with a prolonged drug holiday should be considered.
Different BPs should influence drug holiday decisions differently. Whilst zoledronic acid confers durable, continued anti‐fracture effects, patients embarking on a drug holiday from risedronate may experience relatively more rapid declines in bone density and rebounds in fracture risk. Longer‐term treatment is therefore required with risedronate and clinicians may consider the possibility of a dose of zoledronic acid in risedronate users to promote durability prior to a holiday.
Hip BMD is a robust predictor of fracture in patients embarking on a BP drug holiday. In particular, patients with a rapidly declining hip bone density or a hip T‐score ≤ −2.5 SD may be at risk of holiday‐related fractures.
Current evidence does not support the use of BTMs in decisions on BP drug holidays. While BTMs correlate with BMD trends during a drug holiday, validation of BTM thresholds or % change that would identify patients at risk of holiday‐related fractures has not been established.
BP drug holidays are not drug retirements. Osteoporosis is a chronic, progressive disease and a period of BP use is not curative. A person's risk of fragility fracture will increase with a longer BP drug holiday and clear parameters triggering re‐initiation of treatment should be decided early on.

Drug holidays were consistently associated with declines in BMD at total hip and femoral neck sites, accompanied by increases in BTMs. Inconsistent changes in the lumbar spine BMD related to time‐dependent degenerative artifact and as such, this site was an unreliable predictor of outcomes. By contrast, total hip BMD predicted fractures during a drug holiday and in those persisting on treatment.^(^ ^65^ ^)^ The clinical utility of BTMs in predicting holiday‐related fractures, or in influencing decisions around drug holidays, was not supported by the evidence base.^(^ ^31^ ^)^ Indirectly, however, changes in BTMs correlated with holiday‐related rates of BMD decline.^(^ ^45^ ^)^ A large population‐based study found BTM testing informed treatment decision‐making and was associated with a reduction in fragility fractures (odds ratio [OR] 0.87; 95% CI, 0.85–0.88).^(^ ^66^ ^)^ Regular BTM monitoring may therefore have a role in guiding treatment and drug holiday decisions, although further evaluation is required.

Not all BPs are equal in their antiresorptive effect or durability. Zoledronic acid, with the highest affinity to bone matrix, exerted durable effects on BMD after 6 years of treatment and 3 years off treatment.^(^ ^28^ ^)^ On the other hand, after a 5‐year drug holiday in alendronate users, hip BMD returned to pretreatment baseline levels without retention of effect. Holiday‐related fractures were more frequent in those previously prescribed oral versus intravenous bisphosphonates.^(^ ^57^ ^)^ Risedronate, with the lowest affinity for bone, was associated with relatively early declines in BMD during a drug holiday (~12 months) despite prolonged treatment durations.^(^ ^38^, ^39^ ^)^ Hip fractures were also reportedly more frequent in the risedronate users versus alendronate users after a 3‐year drug holiday.^(^ ^60^ ^)^ Clinicians may thus consider either a shorter duration of drug holiday in risedronate users or the administration of a longer‐acting BP such as zoledronic acid after a course of risedronate to promote a more durable effect prior to a drug holiday.

The emergence of several real‐world studies has yielded important insights on this issue. Effects of adherence, data on premenopausal women and men, and real‐world outcomes in patients embarking on a purposeful drug holiday are presented in these studies. Three large population studies did not find an increase in fracture risk in patients who were treated for more than 3 to 5 years with high adherence. However, changes in femoral neck and total hip BMD during drug holidays in these studies was consistently associated with an increased risk of fracture.^(^ ^40^, ^48^ ^)^ The predominant group examined in these studies were postmenopausal women. Men and premenopausal women were included in some of these studies, but due to small sample sizes, subgroup analyses were not performed.

**Fig. 2:**
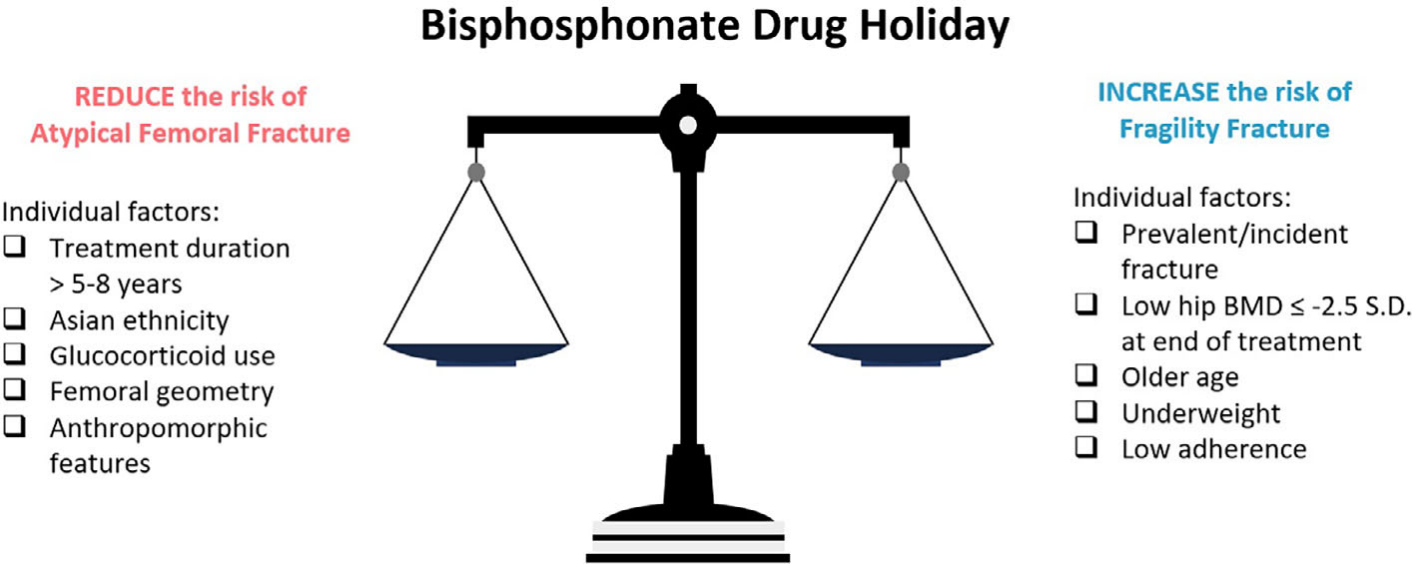
Weighing up the risk of fragility fracture versus AFF.

Identification of candidates suitable for a BP drug holiday requires a personalized assessment of fragility fracture versus atypical fracture risk (Fig. 2). Those >78 years or with a total hip BMD *T*‐score ≤ −2.5 SD with low body weight, poor medication adherence, and prevalent/incident fractures were at risk of holiday‐related fragility fractures in both clinical trials and real‐world settings. FRAX predicted BMD change in patients during BP drug holiday in two studies, but further data are needed before FRAX can be used in the context of holiday decisions.^(^ ^42^, ^67^ ^)^

Studies have not compared the incidence of AFFs in persistent BP users versus fragility fractures in those undertaking a drug holiday *in the same cohort*. The risk of AFF clearly increases with BP treatment duration with an HR increasing from 8.9 (95% CI, 2.8–28) to 43.5 (95% CI, 13.7–138.1) in patients treated for 3 to 5 years versus >8 years.^(^ ^25^ ^)^ Specific risk factors that heighten this risk include Asian ethnicity (HR 4.84; 95% CI, 3.57–6.56), certain anthropomorphic parameters including shorter stature and increased weight, individual femoral geometry, glucocorticoid use, active rheumatoid arthritis, vitamin D deficiency, and a history of minimal‐trauma fracture.^(^ ^25^, ^68^, ^69^, ^70^ ^)^ The specific BP therapy is also relevant, with alendronate users displaying significantly higher risk of AFF than risedronate users in a Swedish cohort (RR 1.9; 95% CI, 1.1–3.3).^(^ ^71^ ^)^

In considering these factors, when is the time right then for a BP drug holiday? A recent study adopted a novel approach in answering this question. Using machine‐learning models, 8 years was the “sweet spot” in which oral BP users optimized their bone quality while avoiding abnormalities in mineralization that would predispose to AFF beyond this time period.^(^ ^20^ ^)^ Drug holidays are effective in neutralizing the risk of AFF with a 48% reduction at 3 to 15 months and further risk reduction up to 79% in the subsequent years following BP discontinuation.^(^ ^25^ ^)^ The notion of “resetting” the risk of AFF by embarking on a drug holiday in at‐risk individuals should however be weighed up against the progressive risk of fragility fracture with a lengthy holiday.^(^ ^25^, ^72^ ^)^ Figure 2 summarizes the various individual patient and treatment factors that clinicians should consider when weighing these competing risks.

Although it is important to consider a drug holiday in the right patient and at the right time, clinicians should be mindful that this does not equate to a “drug retirement.” A clearly documented plan to monitor the patient's bone density and fracture risk during drug holiday is essential, with clear parameters and time points for drug reinitiation. Although BPs are highly effective drugs, durable in their anti‐resorptive effect for years after their use, clinicians and patients should also remember that osteoporosis is a chronic and progressive disease and the risks posed by fragility fracture in older age are formidable.

## AUTHOR CONTRIBUTIONS

**Mawson Wang:** Conceptualization; visualization; writing – original draft; writing – review and editing. **Yu‐Fang Wu:** Conceptualization; formal analysis; writing – original draft; writing – review and editing. **Christian Girgis:** Conceptualization; formal analysis; methodology; project administration; writing – original draft; writing – review and editing.

## Conflicts of Interest

The authors have no conflicts of interest to report.

### PEER REVIEW

The peer review history for this article is available at https://publons.com/publon/10.1002/jbm4.10629.

## References

[1] Sozen T , Ozisik L , Calik BN . An overview and management of osteoporosis. Eur J Rheumatol. 2017;4(1):46‐56. 28293453 10.5152/eurjrheum.2016.048 PMC5335887

[2] McClung M , Harris ST , Miller PD , et al. Bisphosphonate therapy for osteoporosis: benefits, risks, and drug holiday. Am J Med. 2013;126(1):13‐20. 23177553 10.1016/j.amjmed.2012.06.023

[3] Black DM , Abrahamsen B , Bouxsein ML , Einhorn T , Napoli N . Atypical femur fractures: review of epidemiology, relationship to bisphosphonates, prevention, and clinical management. Endocr Rev. 2019;40(2):333‐368. 30169557 10.1210/er.2018-00001

[4] Center JR , Lyles KW , Bliuc D . Bisphosphonates and lifespan. Bone. 2020;141:115566. 32745686 10.1016/j.bone.2020.115566

[5] Reid IR , Horne AM , Mihov B , et al. Fracture prevention with Zoledronate in older women with osteopenia. N Engl J Med. 2018;379(25):2407‐2416. 30575489 10.1056/NEJMoa1808082

[6] Rogers MJ , Mönkkönen J , Munoz MA . Molecular mechanisms of action of bisphosphonates and new insights into their effects outside the skeleton. Bone. 2020;139:115493. 32569873 10.1016/j.bone.2020.115493

[7] Drake MT , Clarke BL , Khosla S . Bisphosphonates: mechanism of action and role in clinical practice. Mayo Clin Proc. 2008;83(9):1032‐1045. 18775204 10.4065/83.9.1032 PMC2667901

[8] Green JR . Bisphosphonates: preclinical review. Oncologist. 2004;9(Suppl 4):3‐13. 10.1634/theoncologist.9-90004-3 15459425

[9] Vitté C , Fleisch H , Guenther HL . Bisphosphonates induce osteoblasts to secrete an inhibitor of osteoclast‐mediated resorption. Endocrinology. 1996;137(6):2324‐2333. 8641182 10.1210/endo.137.6.8641182

[10] Woo SB , Hellstein JW , Kalmar JR . Narrative [corrected] review: bisphosphonates and osteonecrosis of the jaws. Ann Intern Med. 2006;144(10):753‐761. 16702591 10.7326/0003-4819-144-10-200605160-00009

[11] Coxon FP , Thompson K , Roelofs AJ , Ebetino FH , Rogers MJ . Visualizing mineral binding and uptake of bisphosphonate by osteoclasts and non‐resorbing cells. Bone. 2008;42(5):848‐860. 18325866 10.1016/j.bone.2007.12.225

[12] Russell RG , Watts NB , Ebetino FH , Rogers MJ . Mechanisms of action of bisphosphonates: similarities and differences and their potential influence on clinical efficacy. Osteoporos Int. 2008;19(6):733‐759. 18214569 10.1007/s00198-007-0540-8

[13] Lin JH . Bisphosphonates: a review of their pharmacokinetic properties. Bone. 1996;18(2):75‐85. 8833200 10.1016/8756-3282(95)00445-9

[14] Grey A , Bolland MJ , Horne A , Mihov B , Gamble G , Reid IR . Bone mineral density and Bone turnover 10 years after a single 5 mg dose or two 5‐yearly lower doses of zoledronate in osteopenic older women: an open‐label extension of a randomized controlled trial. J Bone Miner Res. 2022;37(1):3‐11. 34585780 10.1002/jbmr.4453

[15] Hanley DA , Adachi JD , Bell A , Brown V . Denosumab: mechanism of action and clinical outcomes. Int J Clin Pract. 2012;66(12):1139‐1146. 22967310 10.1111/ijcp.12022 PMC3549483

[16] Lamy O , Gonzalez‐Rodriguez E , Stoll D , Hans D , Aubry‐Rozier B . Severe rebound‐associated vertebral fractures after denosumab discontinuation: 9 clinical cases report. J Clin Endocrinol Metab. 2017;102(2):354‐358. 27732330 10.1210/jc.2016-3170

[17] Davidoff DF , Girgis CM . Failure of oral risedronate therapy to prevent spontaneous vertebral fracture in a patient ceasing denosumab: a cautionary case. JBMR Plus. 2020;4(10):e10396. 33103026 10.1002/jbm4.10396 PMC7574702

[18] Haas AV , LeBoff MS . Osteoanabolic agents for osteoporosis. J Endocr Soc. 2018;2(8):922‐932. 30087947 10.1210/js.2018-00118 PMC6065487

[19] Girgis CM , Seibel MJ . Atypical femur fractures: a review of the evidence and its implication to clinical practice. Ther Adv Musculoskelet Dis. 2011;3(6):301‐314. 22870488 10.1177/1759720X11416270 PMC3383496

[20] Malluche HH , Chen J , Lima F , Liu LJ , Monier‐Faugere MC , Pienkowski D . Bone quality and fractures in women with osteoporosis treated with bisphosphonates for 1 to 14 years. JBMR Plus. 2021;5(11):e10549. 34761151 10.1002/jbm4.10549 PMC8567493

[21] Schilcher J , Aspenberg P . Incidence of stress fractures of the femoral shaft in women treated with bisphosphonate. Acta Orthop. 2009;80(4):413‐415. 19568963 10.3109/17453670903139914 PMC2823197

[22] Schilcher J , Michaëlsson K , Aspenberg P . Bisphosphonate use and atypical fractures of the femoral shaft. N Engl J Med. 2011;364(18):1728‐1737. 21542743 10.1056/NEJMoa1010650

[23] Dell RM , Adams AL , Greene DF , et al. Incidence of atypical nontraumatic diaphyseal fractures of the femur. J Bone Miner Res. 2012;27(12):2544‐2550. 22836783 10.1002/jbmr.1719

[24] Meier RP , Perneger TV , Stern R , Rizzoli R , Peter RE . Increasing occurrence of atypical femoral fractures associated with bisphosphonate use. Arch Intern Med. 2012;172(12):930‐936. 22732749 10.1001/archinternmed.2012.1796

[25] Black DM , Geiger EJ , Eastell R , et al. Atypical femur fracture risk versus fragility fracture prevention with bisphosphonates. N Engl J Med. 2020;383(8):743‐753. 32813950 10.1056/NEJMoa1916525 PMC9632334

[26] Khosla S , Burr D , Cauley J , et al. Bisphosphonate‐associated osteonecrosis of the jaw: report of a task force of the American Society for Bone and Mineral Research. J Bone Miner Res. 2007;22(10):1479‐1491. 17663640 10.1359/jbmr.0707onj

[27] Black DM , Reid IR , Boonen S , et al. The effect of 3 versus 6 years of zoledronic acid treatment of osteoporosis: a randomized extension to the HORIZON‐Pivotal Fracture Trial (PFT). J Bone Miner Res. 2012;27(2):243‐254. 22161728 10.1002/jbmr.1494 PMC3427916

[28] Black DM , Reid IR , Cauley JA , et al. The effect of 6 versus 9 years of zoledronic acid treatment in osteoporosis: a randomized second extension to the HORIZON‐Pivotal Fracture Trial (PFT). J Bone Miner Res. 2015;30(5):934‐944. 25545380 10.1002/jbmr.2442

[29] Black DM , Schwartz AV , Ensrud KE , et al. Effects of continuing or stopping alendronate after 5 years of treatment: the Fracture Intervention Trial Long‐term Extension (FLEX): a randomized trial. JAMA. 2006;296(24):2927‐2938. 17190893 10.1001/jama.296.24.2927

[30] Ensrud KE , Barrett‐Connor EL , Schwartz A , et al. Randomized trial of effect of alendronate continuation versus discontinuation in women with low BMD: results from the Fracture Intervention Trial long‐term extension. J Bone Miner Res. 2004;19(8):1259‐1269. 15231012 10.1359/JBMR.040326

[31] Bauer DC , Schwartz A , Palermo L , et al. Fracture prediction after discontinuation of 4 to 5 years of alendronate therapy: the FLEX study. JAMA Intern Med. 2014;174(7):1126‐1134. 24798675 10.1001/jamainternmed.2014.1232 PMC4409325

[32] Miller PD , Bolognese MA , Lewiecki EM , et al. Effect of denosumab on bone density and turnover in postmenopausal women with low bone mass after long‐term continued, discontinued, and restarting of therapy: a randomized blinded phase 2 clinical trial. Bone. 2008;43(2):222‐229. 18539106 10.1016/j.bone.2008.04.007

[33] Stock JL , Bell NH , Chesnut CH 3rd , et al. Increments in bone mineral density of the lumbar spine and hip and suppression of bone turnover are maintained after discontinuation of alendronate in postmenopausal women. Am J Med. 1997;103(4):291‐297. 9382121 10.1016/s0002-9343(97)00130-7

[34] Wasnich RD , Bagger YZ , Hosking DJ , et al. Changes in bone density and turnover after alendronate or estrogen withdrawal. Menopause. 2004;11(6 Pt 1):622‐630. 15545790 10.1097/01.gme.0000123641.76105.b5

[35] Tonino RP , Meunier PJ , Emkey R , et al. Skeletal benefits of alendronate: 7‐year treatment of postmenopausal osteoporotic women. Phase III Osteoporosis Treatment Study Group. J Clin Endocrinol Metab. 2000;85(9):3109‐3115. 10999794 10.1210/jcem.85.9.6777

[36] Bone HG , Hosking D , Devogelaer JP , et al. Ten years' experience with alendronate for osteoporosis in postmenopausal women. N Engl J Med. 2004;350(12):1189‐1199. 15028823 10.1056/NEJMoa030897

[37] Ravn P , Weiss SR , Rodriguez‐Portales JA , et al. Alendronate in early postmenopausal women: effects on bone mass during long‐term treatment and after withdrawal. Alendronate Osteoporosis Prevention Study Group. J Clin Endocrinol Metab. 2000;85(4):1492‐1497. 10770187 10.1210/jcem.85.4.6549

[38] Watts NB , Chines A , Olszynski WP , et al. Fracture risk remains reduced one year after discontinuation of risedronate. Osteoporos Int. 2008;19(3):365‐372. 17938986 10.1007/s00198-007-0460-7

[39] Eastell R , Hannon RA , Wenderoth D , Rodriguez‐Moreno J , Sawicki A . Effect of stopping risedronate after long‐term treatment on bone turnover. J Clin Endocrinol Metab. 2011;96(11):3367‐3373. 21865359 10.1210/jc.2011-0412 PMC3205892

[40] Cosman F , Cauley JA , Eastell R , et al. Reassessment of fracture risk in women after 3 years of treatment with zoledronic acid: when is it reasonable to discontinue treatment? J Clin Endocrinol Metab. 2014;99(12):4546‐4554. 25215556 10.1210/jc.2014-1971

[41] Schwartz AV , Bauer DC , Cummings SR , et al. Efficacy of continued alendronate for fractures in women with and without prevalent vertebral fracture: the FLEX trial. J Bone Miner Res. 2010;25(5):976‐982. 20200926 10.1002/jbmr.11

[42] Roberts J , Castro C , Moore AE , Fogelman I , Hampson G . Changes in bone mineral density and bone turnover in patients on 'drug holiday' following bisphosphonate therapy: real‐life clinic setting. Clin Endocrinol (Oxf). 2016;84(4):509‐515. 26715263 10.1111/cen.13012

[43] Curtis JR , Westfall AO , Cheng H , Delzell E , Saag KG . Risk of hip fracture after bisphosphonate discontinuation: implications for a drug holiday. Osteoporos Int. 2008;19(11):1613‐1620. 18483689 10.1007/s00198-008-0604-4 PMC2574626

[44] Adams AL , Adams JL , Raebel MA , et al. Bisphosphonate drug holiday and fracture risk: a population‐based cohort study. J Bone Miner Res. 2018;33(7):1252‐1259. 29529334 10.1002/jbmr.3420

[45] Sølling AS , Christensen DH , Darvalics B , Harsløf T , Thomsen RW , Langdahl B . Fracture rates in patients discontinuing alendronate treatment in real life: a population‐based cohort study. Osteoporos Int. 2021;32(6):1103‐1115. 33411002 10.1007/s00198-020-05745-x

[46] Chiha M , Myers LE , Ball CA , Sinacore JM , Camacho PM . Long‐term follow‐up of patients on drug holiday from bisphosphonates: real‐world setting. Endocr Pract. 2013;19(6):989‐994. 24013976 10.4158/EP12425.OR

[47] Gallagher AM , Rietbrock S , Olson M , van Staa TP . Fracture outcomes related to persistence and compliance with oral bisphosphonates. J Bone Miner Res. 2008;23(10):1569‐1575. 18505366 10.1359/jbmr.080510

[48] Bindon B , Adams W , Balasubramanian N , Sandhu J , Camacho P . Osteoporotic fractures during bisphosphonate drug holiday. Endocr Pract. 2018;24(2):163‐169. 29144808 10.4158/EP171975.OR

[49] Xu LH , Adams‐Huet B , Poindexter JR , Maalouf NM . Determinants of change in bone mineral density and fracture risk during bisphosphonate holiday. Osteoporos Int. 2016;27(5):1701‐1708. 26642963 10.1007/s00198-015-3447-9

[50] Pfeilschifter J , Steinebach I , Trampisch HJ , Rudolf H . Bisphosphonate drug holidays: risk of fractures and mortality in a prospective cohort study. Bone. 2020;138:115431. 32422299 10.1016/j.bone.2020.115431

[51] Statham L , Abdy S , Aspray TJ . Can bone turnover markers help to define the suitability and duration of bisphosphonate drug holidays? Drugs Context. 2020;9:2020‐1‐3. 10.7573/dic.2020-1-3 PMC7216784 32426015

[52] Sølling AS , Harsløf T , Bruun NH , Langdahl B . The predictive value of bone turnover markers during discontinuation of alendronate: the PROSA study. Osteoporos Int. 2021;32(8):1557‐1566. 33517477 10.1007/s00198-021-05835-4

[53] Liel Y , Plakht Y , Tailakh MA . Bone turnover in osteoporotic women during long‐term oral bisphosphonates treatment: implications for treatment failure and “drug holiday” in the real world. Endocr Pract. 2017;23(7):787‐793. 28448762 10.4158/EP171781.OR

[54] Aboughanima A . Risk of spontaneous vertebral fracture during bisphosphonates drug holiday. Egypt Rheumatol Rehabil. 2020;47(1):1‐8.

[55] Bagger YZ , Tankó LB , Alexandersen P , Ravn P , Christiansen C . Alendronate has a residual effect on bone mass in postmenopausal Danish women up to 7 years after treatment withdrawal. Bone. 2003;33(3):301‐307. 13678770 10.1016/s8756-3282(03)00112-1

[56] Fu SH , Wang CY , Hung CC , et al. Increased fracture risk after discontinuation of anti‐osteoporosis medications among hip fracture patients: a population‐based cohort study. J Intern Med. 2021;290(6):1194‐1205. 34237171 10.1111/joim.13354

[57] Curtis JR , Saag KG , Arora T , et al. Duration of bisphosphonate drug holidays and associated fracture risk. Med Care. 2020;58(5):419‐426. 31985584 10.1097/MLR.0000000000001294 PMC8287838

[58] Curtis JR , Chen R , Li Z , et al. The impact of the duration of bisphosphonate drug holidays on hip fracture rates [abstract]. 2017 ACR/ARHP Annual Meeting. San Diego, CA: Annals of the Rheumatic Diseases; 2017. Abstract number 828.

[59] Mignot MA , Taisne N , Legroux I , Cortet B , Paccou J . Bisphosphonate drug holidays in postmenopausal osteoporosis: effect on clinical fracture risk. Osteoporos Int. 2017;28(12):3431‐3438. 28875236 10.1007/s00198-017-4215-9

[60] Hayes KN , Brown KA , Cheung AM , Kim SA , Juurlink DN , Cadarette SM . Comparative fracture risk during osteoporosis drug holidays after long‐term Risedronate versus alendronate therapy: a propensity score‐matched cohort study. Ann Intern Med. 2022;175(3):335‐343. 35007149 10.7326/M21-2512

[61] Cosman F , de Beur SJ , LeBoff MS , et al. Clinician's guide to prevention and treatment of osteoporosis. Osteoporos Int. 2014;25(10):2359‐2381. 25182228 10.1007/s00198-014-2794-2 PMC4176573

[62] Adler RA , El‐Hajj Fuleihan G , Bauer DC , et al. Managing osteoporosis in patients on long‐term bisphosphonate treatment: report of a task force of the American Society for Bone and Mineral Research. J Bone Miner Res. 2016;31(1):16‐35. 26350171 10.1002/jbmr.2708 PMC4906542

[63] Nayak S , Greenspan SL . A systematic review and meta‐analysis of the effect of bisphosphonate drug holidays on bone mineral density and osteoporotic fracture risk. Osteoporos Int. 2019;30(4):705‐720. 30623214 10.1007/s00198-018-4791-3 PMC6499675

[64] Dennison EM , Cooper C , Kanis JA , et al. Fracture risk following intermission of osteoporosis therapy. Osteoporos Int. 2019;30(9):1733‐1743. 31175404 10.1007/s00198-019-05002-w

[65] Banefelt J , Timoshanko J , Söreskog E , et al. Total hip Bone mineral density as an indicator of fracture risk in bisphosphonate‐treated patients in a real‐world setting. J Bone Miner Res. 2022;37(1):52‐58. 34585781 10.1002/jbmr.4448 PMC9298264

[66] Lane NE , Saag K , O'Neill TJ , et al. Real‐world bone turnover marker use: impact on treatment decisions and fracture. Osteoporos Int. 2021;32(5):831‐840. 33236195 10.1007/s00198-020-05734-0 PMC8043891

[67] Leslie WD , Aubry‐Rozier B , Lamy O , Hans D , Manitoba Bone Density Program . TBS (trabecular bone score) and diabetes‐related fracture risk. J Clin Endocrinol Metab. 2013;98(2):602‐609. 23341489 10.1210/jc.2012-3118

[68] Girgis CM , Sher D , Seibel MJ . Atypical femoral fractures and bisphosphonate use. N Engl J Med. 2010;362(19):1848‐1849. 20463351 10.1056/NEJMc0910389

[69] Crouch G , Dhanekula ND , Byth K , et al. The Sydney AFF Score: a simple tool to distinguish females presenting with atypical femur fractures versus typical femur fractures. J Bone Miner Res. 2021;36(5):910‐920. 33528853 10.1002/jbmr.4255

[70] Dhanekula ND , Crouch G , Byth K , et al. Asian ethnicity and femoral geometry in atypical femur fractures: independent or inter‐dependent risk factors? JBMR Plus. 2022;6(4):e10607. 35434447 10.1002/jbm4.10607 PMC9009102

[71] Schilcher J , Koeppen V , Aspenberg P , Michaëlsson K . Risk of atypical femoral fracture during and after bisphosphonate use. Acta Orthop. 2015;86(1):100‐107. 25582459 10.3109/17453674.2015.1004149 PMC4366670

[72] Bégin MJ , Audet MC , Chevalley T , et al. Fracture risk following an atypical femoral fracture. J Bone Miner Res. 2022;37(1):87‐94. 34668223 10.1002/jbmr.4461 PMC9298806

